# Advance directive: does the GP know and address what the patient wants? Advance directive in primary care

**DOI:** 10.1186/s12910-018-0305-2

**Published:** 2018-06-11

**Authors:** Guda Scholten, Sofie Bourguignon, Anthony Delanote, Bieke Vermeulen, Geert Van Boxem, Birgitte Schoenmakers

**Affiliations:** Department of Public Health and Primary Care, University of Leuven, Kapucijnenvoer 33, block J, Box 7001, 3000 Leuven, Belgium

**Keywords:** Primary care, General practice, Advance care planning, Living will

## Abstract

**Background:**

Due to the rapid changes in the medical world and the aging population, the need for advanced care planning grows.

Despite efforts to make this topic discussed, only a minority of patients discusses the advance directive with their general practitioner (GP). This study aimed to map thresholds: What barriers are identified by GPs and patients in preparing and discussing an advance directive?

**Methods:**

A cross section survey in patients and GP’s was performed. Citizens were recruited by multimedia and by street interviews. GP’s were recruited by mailing.

**Results:**

Most of the 502 citizens already heard of an advance directive but only 17 had declared one while 21 never want one. Eighty percent wants to take the initiative themselves but half of the participants wants the GP to be actively involved. Thirty percent finds the document too difficult to understand. The need to draw an advance directive grew with increasing age. Of the 117 GP’s involved, 65% drafted five or less advance directives the past year. A lack of time, experience and a poor access to the correct administrative requirements were only a few of the barriers.

**Conclusions:**

Preparing and drafting an advance directive is a time-consuming and difficult procedure.

Patients and GP’s have the right to be informed and instructed on how to prepare an advance directive.

## Background

Due to the rapidly changing knowledge and technology in the medical world and the aging population, the need for advanced care planning (ACP) for elderly grows. In Belgium 9% of the population dies a sudden death, while 91% dies of prolonged disease [1]. In this latter group, 25% dies of cancer [2, 3]. These numbers are comparable among all developed countries [4, 5]. Clinicians are therefore more than ever confronted with complicated end of life decisions in patients who are unable to actively participate in the decision process [6–9]. Beside, proxies might in best cases not share perspectives and in worse cases have conflicting interests in end of life decisions addressing their ill, care needing relative [10].

In the last phase of life, patients and caregivers often face ethical dilemmas [9, 11, 12]. Palliative care and safeguarding the quality of life are in well selected cases and in consent with the patient are preferable to extensive treatment and life-prolonging operations [5]. An advance directive can be an important tool for organizing end of life care [6, 7, 13]. Advance care planning and drafting an advance directive can alleviate suffering, improve quality of life and support the decision making in end of life care [13–16]. Moreover, ACP does not only serve the patient but will also positively affect the feelings of the proxies and decrease health care costs [9, 16].

Although most people strongly express the wish to be cared for or die at home, only a small minority confirmed this wish in an advance directive [12, 17, 18]. Even when severely or terminally ill, less than 50% of the patients have an advance directive [18, 19]. Moreover, the majority of doctors are unaware of the availability of an advance directive made by their patients [12, 15]. These observations are in contrast with the wish of many patients to enhance quality of life rather than prolong it: adding life to years instead of adding years to life [16].

In Belgium and the Netherlands only a quarter of the seriously ill patients discusses with their general practitioner (GP) a negative advance directive and very few (3.6%) effectively prepared one [2, 3]. The declaration of an advance directive consists of two options: a negative and a positive will. The negative advance directive is legally binding and is applied only when the patient is incapable of giving consent (Law on patient rights, Art. 8 § 4). The use of this document leads to the withdrawal or limitations of medical actions in patients who declared the refusal of certain interventions. A positive declaration of intent is not legally regulated: patients never can impose treatments, interventions or investigations, they can only express their wishes by writing them down.

Both GP’s and patients encounter many barriers to discuss this topic [13, 15, 19]. GP’s do not feel competent and lack the time to start the discussion on this topic [17, 18, 20]. Patients feel reluctant to share their concerns about end of life caring when there are no life threatening medical conditions or because they feel embarrassed or ungrateful in front of their proxies. Patients often are only familiar with the declaration of euthanasia and not with the concept of advance directives. They are confused and not rarely afraid of the unmeant consequences or misinterpretations of a living will. Last but not least, there is a collective social denial of end of life issues [12].

Although, discussing end of life caring in the context of advance care planning is commonly considered as appropriate in chronically ill patients, this matter should also be debatable with healthy people [4, 8, 18]. Indeed advance care planning is a dynamic process, requiring insights and perspectives that are likely to change over time. In Flanders, GP’s are supported by guidelines on opening the discussion on advance care planning, on providing information and on guiding them with their patients through this process (https://www.domusmedica.be/documentatie/downloads/praktijkdocumenten/richtlijnen/1010-vroegtijdige-zorgplanning/file.html). An informative website of the Flemish and Federal health care community is available for patients (www.leif.be).

Despite efforts from policymakers, patient and healthcare organizations to make this topic more widely discussed, only a minority discusses and completes this document with their GP.

This research examines the barriers GPs and patients experience in preparing and discussing an advance directive (advance directive).

## Method

What barriers are identified by GPs and patients in preparing and discussing an advance directive? This quantitative study was conducted with a cross-section of citizens over 65 years and a cross-section of GPs working in Flanders.

### Population and design of citizen survey

The target group consisted of people over 64 years. The only explicit exclusion criterion was age under 64 which was questioned at the start of the survey. An implicit exclusion criterion was as sufficient mastery of the Dutch language. People were approached in public area, by electronic survey, on patient platforms (Alzheimer’s League) and via senior organizations across Flanders. For recruitment via the Alzheimer League, only people with mild cognitive deficits and preserved decision capacity were approached. Surveys were offered both electronically and on paper to keep the participation barrier low. People were free to choose between both strategies. People were informed in writing about the purpose of the study and gave their verbal consent in case of the paper version and by ticking the box in case of the electronic version.

The questions addressed the following outcomes: notion of an advance directive, a need for an advance directive and reasons why no advance directive was drafted yet.

The questionnaire was designed in a quantitative construction and consisted of multiple choice answers. The questionnaire was introduced by a brief explanation of advanced care planning and advance directive. The questionnaire consisted of six questions with Likert scale answer options (from 1 to 4): Have you ever heard of an advance directive? Do you want an advance directive? If you do not already have an advance directive, what is the reason? Who do you expect to take initiative? What do you expect from your doctor/GP? One question was formulated as multi-select: how do you want your GP to involve you in the process. These questions were drafted in accordance with the existing evidence on unmet needs in ACP and compiled into a questionnaire, after researchers and expert consent. Between January 17, 2016 and March 7, 2016 data were collected.

### Population and design GP survey

GP’s were recruited over the Flemish region and without exclusion criteria. The survey was offered by means of an electronic interface. GP’s participated after ‘written’ consent by ticking an agree-box.

The questionnaire for GP’s investigated how many advance directives were drawn up annually, the approach, the initiator of the conversation on the topic and the perceived barriers. The questionnaire was composed by multiple choice answers and free text fields. The questionnaire consisted of four questions in answer to the research question: How many advance directives did you prepare for your patients? What was your approach? What barriers did you encounter in preparing advance directive? How was the conversation on this topic initiated?

Between January 18, 2016 and March 1, 2016 data were collected.

### Ethical approval

The study protocol was submitted to the University Hospitals of Leuven Medical Ethics Committee and approved in December 2015.

### Statistical analyses

For statistical analysis SAS software, version 9.4, was used.

A frequency descriptive analysis was performed for both citizen and GP-survey. A multivariate analysis was performed using age of the patient as the dependent variable and the variables of the patient survey as independent variables. Via the Logistic procedure, a multivariate analysis was performed. “Have you ever heard of an advance directive?” was used as the dependent variable and “Do you need an advance directive?” and “Who do you expect that the initiative comes?” were used as independent variables.

At the GP-level, the multivariate analysis used the number of advance directives as a dependent variable and the approach and how the conversation was initiated as independent variables. A General Linear Model (GLM) was used.

## Results

### Citizens survey (Table 1)

The study population consisted of 502 people with an average age of 71 years. No participants were younger than 64 years.

**Table 1:** survey of citizens

Age participants (y)	≤64		65–74		75–84		85–94		Total	
	n	%	n	%	n	%	n	%	n	%
Number citizens*	44	8,75	290	57,85	149	29,62	19	3,78	502	100
Male^#^	17	38,64	137	47,08	82	55,03	12	63,16	248	49,40
Female^#^	27	61,36	153	52,92	67	44,97	7	36,84	255	50,79
Heard^#^ No	4	9,09	27	9,28	16	10,74	1	5,26	48	9,56
Yes	40	90,91	263	90,72	133	89,26	18	94,74	455	90,63
Need^#^ Yes, I have one	7	15,91	46	15,91	29	19,46	5	25,32	87	17,33
Yes, I want one	16	36,36	105	36,33	51	34,23	2	10,53	176	35,05
No, not now	19	43,18	132	45,67	61	40,94	7	36,84	219	43,62
No, never	2	4,55	6	2,07	8	5,37	5	26,32	21	4,18
Initiator^#^ Me	39	88,64	237	82,00	107	71,81	15	79,95	398	79,28
GP	4	9,09	28	9,68	22	14,77	0	0,00	54	10,75
Family	0	0,00	13	4,49	13	8,72	0	0,00	26	5,17
Other	1	2,27	11	3,80	7	4,70	4	21,05	24	4,78
Task^#,!^ Bring up the subject	14	24,56	79	22,19	49	26,06	2	9,52	144	23,15
GP Explain	19	33,33	131	36,79	59	31,38	6	28,57	214	34,40
Make one	13	22,80	67	18,82	33	17,55	2	9,52	116	18,64
None	5	8,77	37	10,39	24	12,76	4	19,04	70	11,25
Other	6	10,52	42	11,79	23	12,23	7	33,33	78	12,54
Reasons^#,!^										
I don’t think it’s necessary	2	4,34	15	4,91	14	7,77	2	14,28	33	6,05
I’ve never thought about it	9	19,56	48	15,73	28	15,55	4	28,57	89	16,33
It’s too early	6	13,04	71	23,27	17	9,44	1	7,14	95	17,43
I don’t know what it includes	8	17,39	43	14,09	27	15,00	0	0,00	78	14,31
I don’t dare to talk about it	1	2,17	3	0,98	7	3,88	0	0,00	11	2,01
I don’t know what my GP thinks about it	5	10,86	26	8,52	24	13,33	0	0,00	55	10,09
My family doesn’t agree	0	0,00	3	0,98	2	1,11	0	0,00	5	0,91
It contradicts my faith/world-view	1	2,17	6	1,96	4	2,22	3	21,42	14	2,56
I find it hard to make a choice	8	17,39	51	16,72	40	22,22	2	14,28	101	18,53
I find it hard to share my opinion	0	0,00	0	0,00	0	0,00	0	0,00	0	0,00
Other	6	13,04	39	12,78	17	9,44	2	14,28	64	11,74

*This percentage is calculated in proportion to the total number of participants

# These percentages are calculated in proportion to the number of participants in the group, the groups are divided by the number of advance directives. Due to rounding the sum is not always 100%.

! The participants could choose multiple answers, the sum of the answers is therefore not matching the number of participants

Four hundred fifty five people (90.63%) had heard of an advance directive.

Eighty seven (17.33%) citizens had an advance directive, 176 (35.05%) wanted to have an advance directive, 219 (43.62%) citizens wanted one but not at the moment, and 21 (4 18%) citizens never wanted an advance directive.

Fifty four (10.75%) citizens want the GP to be the initiator, 398 (79.28%) citizens want to take initiative themselves and 26 (5.17%) citizens want their family to take the initiative.

One hundred fifty four (23.15%) citizens wanted GPs to raise the issue and 214 (34.40%) citizens wanted the doctor to explain the document. Only 116 (18.64%) citizens wanted the doctor to make up the document and 70 (11.52%) citizens want their GP not to be involved.

One hundred one (18.53%) citizens had no advance directive because they thought the different options were too difficult to interpret or consider. 89 (16.33%) citizens simply never thought about an advance directive. 78 (14.31%) citizens did not know what the document stands for and therefore they had no advance directive.

In the age group 65–74 years 71 (23.27%) citizens thought it was too early to draw up an advance directive. In the 85–94 year group, three citizens stated that their religion / philosophy did not allow the drawing of an advance directive.

The need for an advance directive was significantly correlated with age (F value = 6.43; Pr > F = 0.0003). Citizens, who expected the doctor to take initiative to draw up an advance directive, were 12 times more likely to know the document than people who did not want their GP to take initiative. Citizens who wanted to take initiative were not more familiar with an advance directive than citizens who wanted others to take initiative (OR 3.3, 95% confidence interval [0.357 to 30.368]).

### GP survey (Table 2)

A total of 117 physicians participated in the survey. Four general practitioners did not draft an advanced directive in 2015. Seventy seven GPs (65.81%) drafted five or less advance directives and six GPs (5.13%) drafted more than 15 directives. The number of advance directives drafted by GPs didn’t influence their approach towards patients. Seventy three GPs (62.40%) provided information and planned a follow-up appointment. Seventeen GPs (14.53%) drafted an advance directive during the same consultation the patient mentioned the subject. Ninety five GPs (58.64%) stated that the patient took initiative to draft an advance directive.

**Table 2:** Survey General Practitioners (GPs)

Number advanced directives	≤5		6–10		11–15		> 15		Total	
	n	%	n	%	n	%	n	%	n	%
Number GPs*	77	65,81	20	17,09	14	11,97	6	5,13	117	100
Approach^#^										
During the same consultation	14	18,18	2	10,00	0	0,00	1	16,67	17	14,53
A different consultation was made	7	9,09	2	10,00	1	7,14	1	16,67	11	9,40
I gave information and planned a follow-up consultation	45	58,44	13	65,00	11	78,57	4	66,67	73	62,40
Other	11	14,29	3	15,00	2	14,29	0	0,00	16	13,68
Initiator^#,!^										
I brought up the subject	18	17,47	7	24,13	7	29,16	0	0,00	32	19,75
The patient brought up the subject	63	6,11	15	51,72	11	45,83	6	100	95	58,64
A family-member brought up the subject	16	15,53	3	10,34	3	12,50	0	0,00	22	13,58
Other	6	5,82	4	13,79	3	12,50	0	0,00	13	8,02
Obstacles^#,!^										
It’s very time-consuming	31	22,46	8	29,62	7	25,00	0	0,00	46	23,11
The form is too difficult	18	13,80	2	7,40	4	14,28	3	50,00	27	13,56
Lack of knowledge	15	10,86	4	14,81	1	3,57	1	16,66	21	10,55
Lack of experience	27	19,56	3	11,11	1	3,57	0	0,00	31	15,57
Bad experience	1	0,72	0	0,00	0	0,00	0	0,00	1	0,50
No classification number	10	7,24	4	14,81	3	10,71	0	0,00	17	8,54
Heavy/emotional subject	16	11,59	3	11,11	6	21,42	2	33,33	27	13,56
Other	20	14,49	3	11,11	6	21,42	0	0,00	29	14,57

*This percentage is calculated in proportion to the total number of participants

# These percentages are calculated in proportion to the number of participants in the group, the groups are divided by the number of advance directives. Due to rounding the sum is not always 100%.

! The participants could choose multiple answers, the sum of the answers is therefore not matching the number of

The barriers cited by the GPs in preparing an advance directive were mainly categorized as: “It is time-consuming” (23.11%, *N* = 46), “Lack of experience” (15.57%, *N* = 31) and “The application is too difficult/complex” (13.56%, *N* = 27), “Too loaded/emotional” (13.56%, *N* = 27), “Lack of knowledge” (10.55%, *N* = 21) and “No classification number” (8.54%; *N* = 17).

The multivariate analysis with the number of advanced directives as independent variable and the approach and the initiator as dependent variables showed that the approach (*F* value = 0.37, Pr > *F* = 0.7738) as well as the initiator of the subject (*F* value = 0.65, Pr > *F* = 0.5841) did not significantly influenced the number of advance directives drafted up by the GP.

## Discussion

This study shows that the majority of the citizens already heard about an advance directive. Despite this, more than half of the surveyed GPs made 5 or less advance directives last year. These observations confirm previous findings regarding the (low) number of signed advance directives [1, 18, 21, 22].

Most GP’s provide information and schedule a follow-up consultation in agreement with the Flemish guideline for drafting advance directives. The main barrier remains the time-consuming impact of these appointments. Other authors suggest to provide the preparatory instructions and information by para-medical healthcare worker to overcome these thresholds [8, 12, 23]. Social workers could explain the legislation, exemplify the options and inform about the role of representatives [21]. Explaining and supporting medical decisions, considering diagnosis and prognosis and meeting the values of the patient, remain the task of the physician.

This study shows that citizens expect that the GP plays an informative role and explains what an advance directive stands for. However, the intervention of an external counselor was not further investigated.

GP’s are for evident reasons expected to take the initiative in advance care planning. In this strategy or procedure, the GP shows his respect and his engagement in this matter [24] (http://www.palliatieve.org/). First, the GP should reassure the patient that he engages to take care at the end of life. In contrast, only very few GP’s timely take the initiative in advance care planning and they even feel reluctant to communicate about the end of life. Moreover, doctors often communicate in too optimistic terms about life expectation: to maintain hope in patients but also because accurate prognoses are hard to make. This reality influences the end of life care in a negative way. Although, research demonstrates that patients who have a better awareness and understanding of the topic participate more actively and safely in end of life decisions and care [6, 21]. Second, this strategy brings relatives and patients closer to each other, positively affects the patient-doctor relationship and increases the number of well documented advance directives. GP’s in contrast, are not used to timely take the initiative in advance care planning and they even feel reluctant to communicate about the end of life. This phenomenon often influences the end of life care in a negative way. Although, research demonstrated that a better awareness and understanding in patients help to participate actively and safely in end of life decisions and care. Above, this strategy brings relatives and patient closer to each other, positively affects the patient-doctor relationship and increases the number of documented advance directives.

In the age group 85 to 94 years, a quarter of people never felt the need for an advance directive. This is in contrast with previous research that observed that significantly more elderly requested to declare an advance directive [19, 20]. In this study, the group of 85–94 years could be too small to draw conclusions. Further, drafting an advance directive is motivated by the spiritual and the religious background of the people, which is also confirmed by this study [11, 20, 25].

In contrast to other studies this research shows that the citizens prefer to take initiative themselves in preparing an advance directive [4, 11, 12, 15, 16]. This observation could be explained by a population selection bias. Earlier studies involved an older population (≥ 75 years) and / or only included terminally ill people or people with a particular life threatening disease [11, 15]. A second explanation might be attributed to a Western value: the highly appreciated autonomy and self-determination of the patient [7, 12]. In this societal perspective and supported by the patient empowerment model, patients desire and are encouraged to maintain control over decisions regarding their health. Thirdly, this observation may be due to the participation of a well-informed population. In this study, 90% of the citizens already heard of an advance directive. Though, the citizens were not asked to clarify the concept.

Some GP’s reported that it is time consuming to explain the difference between an advance directive and an intention of euthanasia. Currently, a conversation about ACP takes on average 5.6 min. In general, the doctor claims two thirds of the conversation time [3]. Financial compensation for this type of consultation probably leads to more attention and time for ACP [3]. To inform the public, the government could organize awareness campaigns addressing the topic.

The low number of formally declared living wills and the discrepancy between the expectations of GP’s and patients probably are mainly due to the complexity of the subject [5]. Patients often have little understanding of medical diagnoses, prognosis and interventions. Therefore, it might be too demanding to accurately judge and relate survival to quality of life [24, 26]. It is difficult for patients to estimate the evolution of a disease and it is therefore difficult to determine the appropriate moment to declare an advance directive [10, 14, 16, 26, 27]. Knowledge of the particular legislation is also required to correctly complete the form [10, 16]. Both patients and doctors face these problems. Further training of patients and doctors is required to improve insights in legal, practical and moral issues in the drafting of an advance directive.

This study shows that there is a gap between the actual and the desired number of advance directives. The study of Sahm et al. shows a similar trend [19]. A majority of citizens is interested in discussing end of life care. Citizens who indicate that an advance directive does not affect them, usually do not reject the application, but rather miss a sense of urgency to create one [28].

Studies show that a conversation about the end of life leads to less invasive therapies, improves quality of life and can even add time to life [26, 29]. Conversations about the end of life also lead to a better grieving process and less stress and anxiety among family members. Finally, an advance directive is not the end point of advanced care planning [16]. For many patients, the hypothetical situation of end of life does not correspond to reality [12]. Declaring an advance directive helps people to express their values, goals and preferences. An advance directive can be considered as the framework to facilitate a conversation about the end of life with others [16, 27].

### Strengths and limitations

The major strength of this study is the large sample size and the recruiting of citizens instead of patients. Citizens were recruited on weekly fairs and other public places. This means that we approached a very representative population that is not necessary chronically or terminally ill or particularly interested in end of life care. A limitation of this study lies in the limited response ratio (1/3) among GP’s. Possibly only doctors interested in the subject completed the survey. On the other hand, more than half of these doctors made 5 or less advance directives in 2015. Additionally, a tenth of the GPs declared that a lack of knowledge was an important threshold. Finally, regarding the patient population, it is very likely that only Dutch speaking or native Flemish citizens were included since a good mastery of Dutch was required to complete the survey.

In the study a quantitative design was chosen intentionally. This strategy permitted to collect sufficient data to detect trends and to draw the gridlines for a qualitative follow up study with in depth interviews and focus group discussion.

## Conclusion

A well-drafted advance directive has a prominent role in ACP and can provide an improvement in the quality of life. Although the public need is high, the number of formally drafted advance directives remains low. This finding is mainly due to the time-consuming process and inherent to the delicate character of end of life conversations. Further research could examine whether the awareness and information process could be a task for other health care workers. Another explanation for the low number of advance directives is the complexity of the actual application. Despite campaigns and local initiatives citizens find the form and procedure hard to understand. Patients and caregivers will certainly benefit from particular consultation rounds about end of life care, analogous to the attention and time spent on prevention.

## Abbreviations

ACP: Advance Care Planning
AD: Advance Directive
GP: General Practitioner
